# Diagnostic Potential of New Systemic Inflammation Markers (MLR, SIRI, CAR, dNLR, ALB/dNLR) in Predicting the Severity of Pathogenic Signs of Rheumatoid Arthritis

**DOI:** 10.17691/stm2026.18.2.04

**Published:** 2026-04-30

**Authors:** E.G. Pukhaeva, A.K. Badtiev, S.G. Dzgoev, F.E. Salamova, A.V. Alborova

**Affiliations:** Researcher, Laboratory of Subcellular Structures, Department of Molecular and Cellular Mechanisms of Autoimmune Diseases; Institute of Biomedical Investigations, the Affiliate of Vladikavkaz Scientific Center of the Russian Academy of Sciences, 1 Williams St., Mikhailovskoye Village, Prigorodny District, Republic of North Ossetia — Alania, 363110, Russia; PhD, Senior Researcher, Laboratory of Subcellular Structures, Department of Molecular and Cellular Mechanisms of Autoimmune Diseases; Institute of Biomedical Investigations, the Affiliate of Vladikavkaz Scientific Center of the Russian Academy of Sciences, 1 Williams St., Mikhailovskoye Village, Prigorodny District, Republic of North Ossetia — Alania, 363110, Russia; PhD, Researcher, Laboratory of Subcellular Structures, Department of Molecular and Cellular Mechanisms of Autoimmune Diseases; Institute of Biomedical Investigations, the Affiliate of Vladikavkaz Scientific Center of the Russian Academy of Sciences, 1 Williams St., Mikhailovskoye Village, Prigorodny District, Republic of North Ossetia — Alania, 363110, Russia; Researcher, Laboratory of Subcellular Structures, Department of Molecular and Cellular Mechanisms of Autoimmune Diseases; Institute of Biomedical Investigations, the Affiliate of Vladikavkaz Scientific Center of the Russian Academy of Sciences, 1 Williams St., Mikhailovskoye Village, Prigorodny District, Republic of North Ossetia — Alania, 363110, Russia; Senior Laboratory Assistant, Laboratory of Subcellular Structures, Department of Molecular and Cellular Mechanisms of Autoimmune Diseases; Institute of Biomedical Investigations, the Affiliate of Vladikavkaz Scientific Center of the Russian Academy of Sciences, 1 Williams St., Mikhailovskoye Village, Prigorodny District, Republic of North Ossetia — Alania, 363110, Russia

**Keywords:** rheumatoid arthritis, hypoxia resistance, CAR, ALB/dNLR, SIRI, synovitis, connective tissue, inflammation markers

## Abstract

**The aim of the study** was to histologically validate the diagnostic potential of new systemic inflammation biomarkers (MLR, SIRI, CAR, dNLR, ALB/dNLR) in assessing the severity of rheumatoid arthritis (RA) induced in warm-blooded animals with genetically determined resistance to hypoxia.

**Materials and Methods:**

An autoimmune RA model (experimental groups) was induced by subcutaneous injection of complete Freund’s adjuvant into the right hind limbs of 8-month-old male rats of strains with high (HR/SmY) and low (LR/SmY) resistance to hypoxia. Rats in the control groups received only the solvent. After 35 days, blood was collected from the hearts of all the rats. To calculate the new inflammation biomarkers — CAR, ALB/dNLR, SIRI, dNLR, NC/LC, and MLR — the following laboratory parameters were used: ALB (albumin), CRP (C-reactive protein), MC (monocytes), LC (lymphocytes), and NC (neutrophils). The effectiveness of disease severity assessment using these biomarkers was determined based on histological examination of plantar sections of the tarsal and metatarsophalangeal joints of control and experimental rats.

**Results:**

Genetically determined resistance to hypoxia is an important factor in the onset of the pathogenetic RA mechanisms. Low hypoxia resistance was associated with more severe connective tissue pathology compared to high resistance: LR/HR — 164 points/113 points, p≤0.05. The severity of pathomorphological RA changes (considering the organism’s resistance to hypoxia), identified histologically (cartilage degeneration: LR/HR — 29 points/18 points, p≤0.01; general joint inflammation: LR/HR — 37 points/26 points, p≤0.01; osteolysis: LR/HR — 6 points/0 points, p≤0.01), was most effectively reflected by the following integral inflammation indices: CAR (LR/HR — 11.55·10^–6^ units/12.73·10^–6^ units), ALB/dNLR (LR/HR — 86.93 units/78.51 units), and SIRI (LR/HR — 0.14 units/ 0.19 units).

**Conclusion:**

The severity of pathomorphological RA signs depends on genetically determined resistance to hypoxia. For effective prediction of the disease, risk of complications, and treatment efficacy, it is advisable to use the new systemic inflammation biomarkers CAR, ALB/dNLR, and SIRI.

## Introduction

Rheumatoid arthritis (RA) ranks 42^nd^ in the world as a cause of disability, leading to permanent incapacity for work in half of the patients with a disease history of 3–5 years. As a systemic immuno-inflammatory connective tissue disease, RA is characterized by numerous genetic polymorphisms associated with B and T cells and is accompanied by progressive joint destruction and internal organ involvement. A high risk of reduced life expectancy of patients (by an average of 10 years) is predicted due to the development of comorbid conditions [1].

The pathogenetic RA mechanisms are associated with changes in the innate immune reactivity, aberrant reciprocal regulation of T and B cells, an imbalance between the synthesis of pro- and anti-inflammatory cytokines, and polarization of the immune response towards Th1 and Th17 types. The formation of autoantibodies targeting post-translationally modified citrullinated proteins, as well as the induction of autoantibodies specific to carbamylated and acetylated peptides, mediate the development of synovitis. Chemokines produced by macrophages, lymphocytes, connective tissue cells, and epithelial cells promote the migration of immune cells (T lymphocytes, B lymphocytes, plasma cells, macrophages, mast cells, activated stromal cells, and synovial fibroblasts) to inflammation sites, where activated synovial fibroblasts stimulate synovial membrane hyperplasia and pannus formation. The degraded extracellular matrix of inflamed tissue contains infiltrating T and B lymphocytes, plasma cells, mast cells, and macrophages. Overproduction of proinflammatory cytokines (IL-1, IL-6, IL-17, TNF-α) activates chondrocytes, matrix metalloproteinase expression, and degradation of protein components of the extracellular matrix, as well as aggrecanases, therefore stimulating cartilage destruction and increasing osteoclast-mediated bone resorption [2, 3].

As the RA etiology remains unknown, effective etiotropic treatment is currently challenging and represents one of the complex problems of modern clinical medicine and pharmacology that requires a solution. Currently, the response rate to RA treatment regimens rarely exceeds 60%. Studies by Abramkin et al. [4] report that over a five-year treatment period, only 26.5% of patients achieved sustained remission, 44.6% showed a satisfactory response, and 28.9% did not respond to treatment.

Despite the heterogeneity of RA etiology and development mechanisms, as well as the wide spectrum of phenotypes and endotypes, a unifying objective criterion for the pathogenetic relatedness of disease symptoms is the synovial hypoxia [5]. According to Falconer et al. [6], in the RA patient joints, the oxygen tension is more than eight times lower (<1%) than in healthy individuals. Wang et al. [5] reported that oxygen content in synovial fluid samples from the knee joints of RA patients was significantly lower (in some cases, 0 mm Hg) compared to samples from patients with osteoarthritis or traumatic effusions. As a consistent RA feature, the synovial hypoxia contributes to disease progression. Hypoxia-inducible factors directly or indirectly influence each disease pathogenesis component, including oxidative cell damage, immuno-inflammatory processes, angiogenesis, cartilage destruction, bone resorption, and the development of comorbid conditions [7].

Recently, there has been studied a potential of new high-tech diagnostic imaging approaches that assess RA activity by monitoring oxygenation changes in affected synovial tissue. Promising results were published in 2023–2024 by two independent research groups. Using photoacoustic imaging for hypoxia [8] and multimodal magnetic resonance imaging/ultrasound imaging of affected joints [9], researchers found out a correlation between decreased synovial fluid oxygen content, standard clinical parameters, and higher disease activity in RA patients.

It should be noted that hypoxia resistance is a genetically determined individual characteristic that influences physiological mechanisms, as well as the risks of developing and the severity of inflammatory, cardiovascular, and neurodegenerative diseases, partly depending on environmental conditions [10]. According to Zarubina [11], 80–95% of Wistar rats have low tolerance to hypoxia, while the majority of August rats are highly resistant animals.

In the human population, hypoxia resistance is heterogeneous and genetically predetermined [12–15]. Currently, there are no reliable markers of hypoxia resistance available for clinical practice. Preclinical studies have attempted to assess an organism’s tolerance to insufficient oxygenation using indices calculated from standard hematological parameters. Based on these parameters, Kondashevskaya et al. [16] identified that in a population of Wistar rats, 30% were highly resistant to hypoxia (HR), 40% were low-resistant (LR), and 30% were moderately resistant. Therefore, the development of new effective approaches for RA diagnosis and therapy requires considering the complex role of hypoxia and resistance to it in the disease’s pathogenesis.

The American College of Rheumatology (ACR), the European Alliance of Associations for Rheumatology (EULAR), and the Association of Rheumatologists of Russia recommend assessing RA activity using one of the following available indices: DAS28, CDAI, SDAI, RAPID3, or PAS-II. Since the main classification criterion for RA severity is visually confirmed synovial joint inflammation (synovitis), the key indicator of treatment efficacy is the assessment of inflammatory activity changes. The RA classification criteria determining disease activity index include the erythrocyte sedimentation rate (ESR) and the C-reactive protein (CRP) concentration (e.g., DAS28-ESR, DAS28-CRP; SDAI-CRP), which reflect local and systemic inflammatory processes in RA [3].

The advanced imaging methods mentioned above can accurately determine the severity of pathological processes in RA. However, they require significant time and material resources. Moreover, the existing inaccuracies and limitations of standard laboratory markers often inadequately reflect RA activity [17–19]. All of this necessitates the search for reliable and easily calculated biomarkers. Recently, scientific publications [20–29] have suggested that indices of systemic inflammation may enable more effective diagnosis of inflammatory activity compared to conventional laboratory parameters. These systemic inflammation indices (MLR, SIRI, CAR, dNLR, ALB/dNLR) are calculated based on complete blood count parameters.

In contrast to the widely reported research on the role of new systemic inflammation biomarkers in predicting the course of cardiovascular diseases [20–23], studies devoted to the management of autoimmune diseases, including RA, are insufficient [24–28]. Within the Russian literature available to us, clinical studies are limited to data presented by Muravyov et al. [29]. We have not recorded any preclinical studies that histologically confirm or refute the correlation between the severity of synovial joint inflammation and these new systemic inflammation biomarkers.

Given that a personalized approach in healthcare aims to optimize diagnosis, treatment, and disease prevention by taking into account individual patient characteristics, evaluating the role of genetically determined hypoxia resistance is of particular interest.

**The aim of the study** was to histologically validate the diagnostic potential of new systemic inflammation biomarkers (MLR, SIRI, CAR, dNLR, ALB/dNLR) in assessing the severity of rheumatoid arthritis induced in warm-blooded animals with genetically determined resistance to hypoxia.

## Materials and Methods

The study was conducted at the Institute of Biomedical Investigations, the Affiliate of Vladikavkaz Scientific Center of the Russian Academy of Sciences (Russia). The study protocol was approved by the local ethics committee, and it complied with the rules and ethical standards for the care and use of laboratory animals as outlined in the National Research Council guidelines (2011) and GOST 33044-2014 “Principles of Good Laboratory Practice”.

The animals were kept under standard vivarium conditions with a 12-hour light/dark cycle and free access to food and water.

The experiment design was the following. An autoimmune RA model was induced in 8-month-old male rats of the HR/SmY (highly resistant to hypoxia) and LR/SmY (low resistant to hypoxia) strains, bred at the Andreevka branch of the National Research Center for Biomedical Technologies of the Federal Medical and Biological Agency of Russia. Before the study, the rats underwent a 30-day acclimatization period. Four experimental groups were formed from animals weighing 400–450 g (12 rats per group): control group 1 — LR/SmY strain; control group 2 — HR/SmY strain; experimental group 3 — LR/SmY strain; experimental group 4 — HR/SmY strain. Autoimmune RA was induced in rats from the experimental groups. The complete Freund’s adjuvant (Difco Laboratories, USA) was injected subcutaneously into the right hind limb at a dose of 0.1 ml per 200 g of body weight. The rats from the control groups were similarly injected with vaseline oil (liquid paraffin) as a solvent at a dose of 0.1 ml per 200 g of body weight [30]. The study lasted 35 days.

At the end of the experiment, blood was collected from the heart under general anesthesia, then the animals were euthanized in a CO_2_ chamber. Blood samples were analyzed using an Abacus Vet 5 hematology analyzer (Diatron, Austria) and a ChemWell-T biochemical analyzer (Awareness Technology Inc., USA) to determine CRP and albumin concentrations using biochemical reagent kits from Olvex Diagnostikum (Russia). The certified equipment and regular calibration checks ensured the measurement accuracy. The following laboratory parameters were measured to calculate systemic inflammation indices: ALB — serum albumin level (g/L); CRP — C-reactive protein level (g/L); MC — monocyte count (%); LC — lymphocyte count (%); NC — neutrophil count (%).

Formulas for calculating the inflammation indices were the following [31–33].

MLR = MC : LC;

SIRI = NC · MC : LC;

CAR = CRP : ALB;

NC/LC = NC : LC;

dNLR = NC : (LC – NC);

ALB/dNLR = ALB : dNLR.

For histological examination of the tarsal and metatarsophalangeal joints (plantar sections), the hind limbs were amputated at the level of 1/3 of the tibia and placed in 10% neutral buffered formalin. Decalcification was performed for two weeks using an electrolyte solution containing formic and hydrochloric acids. Tissue specimens were processed using standard methods, stained with hematoxylin and eosin, and analyzed under a Primo Star light microscope (ZEISS, Germany) equipped with a ToupCam 9.0 MP digital camera (ToupCam, China) at magnifications of 40×, 100×, 400×, and 1000×. Images were processed using ToupView software.

The severity of RA manifestations was assessed using a scale modified by Hegen [34], as presented in the study by Muzhikyan et al. [35]. Destructive changes in the fibrous and synovial capsules were scored (from 0 to 5) based on the following parameters: presence of infiltrate and exudate, characterizing general joint inflammation; severity of joint space narrowing; degree of osteoclast-mediated bone matrix resorption (osteolysis); and increased cellularity of the synovial membrane (hyperplasia). Cartilage destruction was evaluated based on structural changes in the cartilage surface (irregularities, fissures, and delamination), reduced staining intensity, and the level of chondrocyte proliferative activity (ratio of isogenic chondrocytes to groups of 2–3 or more cells).

### Statistical data analysis

The obtained results were processed and compared using Statistica software (StatSoft, USA). The normality of the distribution was assessed using the Shapiro–Wilk test (n<50). As the distribution deviated from normal, the nonparametric Mann–Whitney U test was used to compare statistical hypotheses. The median frequency of the parameter was presented with the interquartile range, Me [25; 75]. Statistical significance of differences between groups was assessed between control and experimental groups (1/3; 2/4), between control groups (1/2), and between experimental groups (3/4). Differences were considered statistically significant at p≤0.05. The Pearson correlation coefficient (r_xy_) was used to evaluate the relationship between parameters. Based on the r_xy_ value, the correlation strength was determined using the Chaddock scale, and its direction (positive or negative) was noted.

## Results

**Figure 1.**
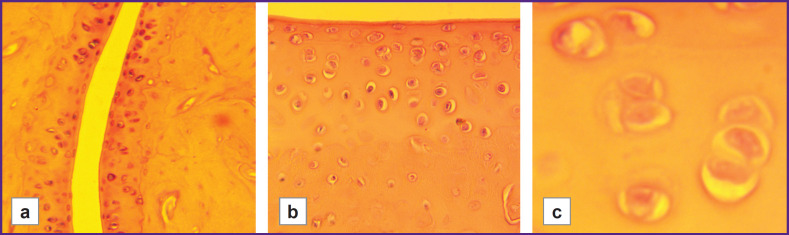
Joint cavity and cartilage tissue in control groups of hypoxia-low-resistant male rats of the LR/SmY strain and hypoxia-high-resistant male rats of the HR/SmY strain: (a) normal joint cavity (LR/SmY; 100×); (b) normal synovial membrane and cartilage tissue, clear borders between layers (HR/SmY; 400×); (c) isogenic groups of chondrocytes (2–3 cells in each) in the mature cartilage zone and normal intercellular matrix (LR/SmY; 1000×)

***In the control groups of rats*** with different hypoxia resistance, the histoarchitecture of the joint tissues of the hind limbs, consisting of metatarsal bones, proximal, middle, and distal phalanges, was examined. No pathological changes were observed in these samples (Figure 1).

***In each experimental group**,* an inflammatory process was observed in the joints — LR/HR=1.4 (p≤0.01) (Table 1).

**T a b l e 1:** Pathomorphological characteristics of adjuvant-induced rheumatoid arthritis in rats with different tolerance to hypoxia (points)

Parameters	Low-resistance to hypoxia animals	High-resistance to hypoxia animals
General joint inflammation (infiltrate, effusion)	37	26**
Severity of joint space narrowing	25	19
Bone tissue changes (osteolysis)	6	0**
Synovial membrane hyperplasia	31	25
Pannus severity (granulation tissue)	36	26
Severity of cartilage degeneration	29	18**
Total group score	164	113*

N o t e: statistical significance of differences in pathomorphological signs of rheumatoid arthritis in animals with different tolerance to hypoxia:

* p≤0.05;

** p≤0.01.

Perivascular infiltrate was represented by lymphocytes and plasma cells. The degree of synovial membrane proliferation and hyperplasia ranged from moderate to severe (LR/HR=1.2). The pannus was formed by proliferating loose granulation tissue, characterized by increased angiogenesis and the presence of macrophages, neutrophils, and eosinophils. In more severe RA forms, pannus invaded into the subintimal layer of the fibrovascular synovial layer with varying severity (LR/HR=1.4). Joint spaces were pathologically narrowed (ranging from minimal to moderate), with signs of deformation and fibrous ankylosis (LR/HR=1.3).

**Figure 2.**
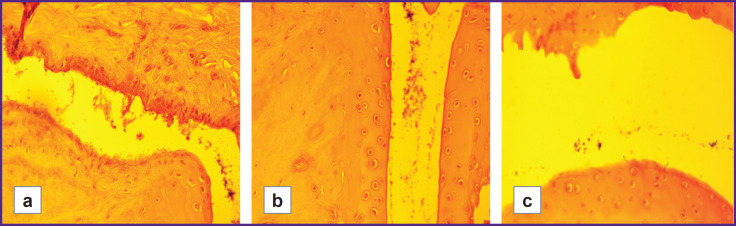
Inflammatory processes in the joint cavity of hypoxia-low-resistant rats with model rheumatoid arthritis (LR/SmY strain): (a) synovial hyperplasia, infiltration by granulation tissue cells (100×); (b) pronounced perivascular infiltrate in the joint cavity (100×); (c) early stages of pannus formation, inflammatory infiltrate in the joint cavity (100×)

**Figure 3.**
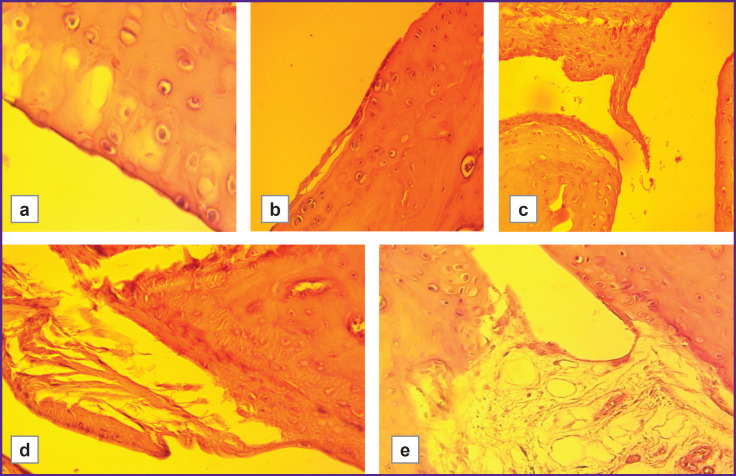
Degenerative cartilage changes in hypoxia-low-resistant rats with model rheumatoid arthritis (LR/SmY strain): (a) minor damage (vacuolization) (400×); (b) superficial erosions and early stages of pannus formation (100×); (c) active synovial cell proliferation, destruction of the superficial layer, cartilage disintegration, mild infiltrate and desquamated fragments of the synovial membrane in the joint cavity, pannus ingrowth into the joint cavity (100×); (d) destruction of the superficial cartilage layer, increased proportion of fibrous connective tissue in the pannus, focal dystrophy and necrosis of chondrocytes (100×); (e) cartilage destruction, loss of clear borders between layers, focal dystrophy and necrosis of chondrocytes, formation of fibrous ankylosis (100×)

**Figure 4.**
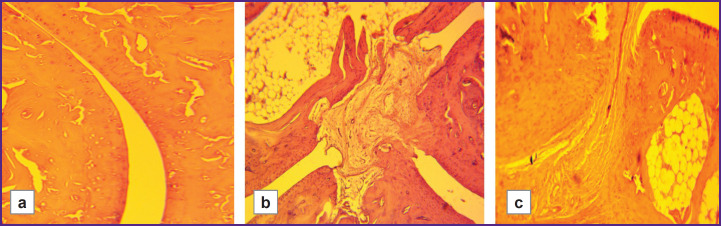
Pathological changes in the joint cavity of hypoxia-low-resistant rats with model rheumatoid arthritis (LR/SmY strain): (a) moderate narrowing of the joint cavity (40×); (b) pronounced pannus overgrowth, formation of erosions and fissures, focal loss of superficial cartilage layers, fibrous ankylosis (40×); (c) critical narrowing of the joint space, leading to severe degenerative cartilage changes (chondrocyte dystrophy and necrosis, loss of the superficial layer, formation of erosions and fissures), ankylosis (40×)

The severity of cartilage degenerative changes was statistically significantly higher in the group with low hypoxia resistance than in the highly resistant group (LR/HR=1.61, p≤0.01). These changes ranged from early stages of granulation tissue replacement by mature fibrous tissue, as well as from superficial erosions and focal matrix vacuolization, to signs of connective tissue disintegration, loosening of the intercellular substance around isogenic chondrocyte groups, formation of erosions and fissures, and the development of ankylosis (Figures 2–4).

**Figure 5.**
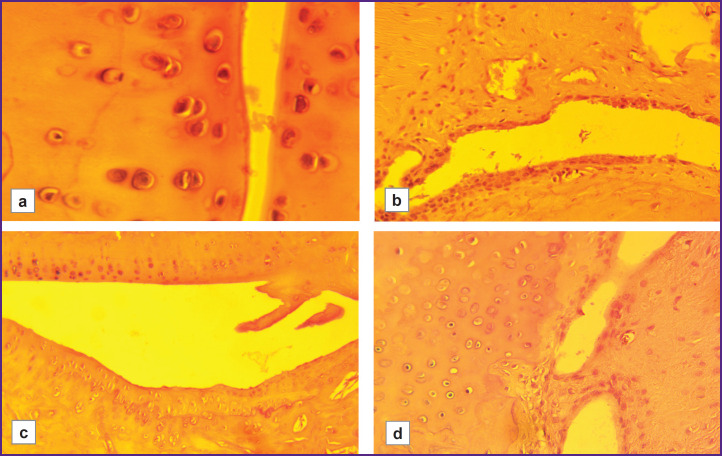
Pathomorphological changes in hypoxia-high-resistant rats with model rheumatoid arthritis (HR/SmY strain): (a) moderate narrowing and desquamated fragments of the synovial membrane in the joint cavity (400×); (b) replacement of the normal synovial membrane by granulation tissue, pannus formation, mild infiltrate and desquamated fragments in the cavity, moderate erosion (100×); (c) pannus formation, mild deformation of the hyaline cartilage (borders between layers remain clearly visible) (100×); (d) ingrowth of new granulation tissue into the synovial cavity, single leukocytes, and desquamated fragments in the joint cavity (100×)

In animals with low resistance to hypoxia, foci of osteoporosis were observed in the presence of more severe degenerative cartilage changes. These foci were not observed in the highly resistant rats (LR/HR — 6 points/0 points). The histoarchitecture of connective tissue was better preserved in animals highly resistant to hypoxia than in those with low resistance (Figure 5). Joint surfaces were mostly smooth, and only a few animals had minor cartilage disintegration and signs of joint space narrowing. Borders between layers were preserved. Chondrocytes in the mature cartilage zone were arranged in regular isogenic groups. Minor fibrous ankylosis was observed in 2 out of 12 animals.

The total group score for the severity of pathomorphological RA signs was 1.5 times higher in the LR rat group than that in the HR animals (p≤0.05). The intragroup histological severity of pathomorphological RA changes was determined based on individual scores for each animal; it is presented in Table 2.

**T a b l e 2:** Intragroup distribution of individual scores for histological severity of pathomorphological signs of rheumatoid arthritis in rats with different tolerance to hypoxia

Experimental group	Severity of pathology (n)		
	Mild (0 to 10 points)	Moderate (11 to 14 points)	Severe (15 to 20 points)
Low-resistant	3 out of 12	3 out of 12	6 out of 12
High-resistant	7 out of 12	5 out of 12	0

The maximum possible disease severity level (critical) for a single animal according to the Hegen–Muzhikyan scale is 30 points. In our study, the maximum severity of RA signs corresponded to the “significant” level and was observed in half of the LR animals (6 out of 12). In the HR group, which had the highest number of animals with mild pathology (7 out of 12), such signs were completely absent.

The diagnostic potential of the new systemic inflammation markers (Table 3) was evaluated based on the histological results shown in Tables 1 and 2.

**T a b l e 3:** Dynamics of laboratory indices in rats with high and low resistance to hypoxia in control groups and in groups with a rheumatoid arthritis model

Indices	Statistical parameters	Low-resistance to hypoxia animals		High-resistance to hypoxia animals	
		Control	Experimental	Control	Experimental
MLR	Me [25; 75]	0.05 [0.02; 0.07]	0.06 [0.05; 0.08]	0.05 [0.04; 0.07]	0.10 [0.07; 0.12]
	p		0.1486*	0.6647***	0.0178** 0.1123****
SIRI	Me [25; 75]	0.07 [0.04; 0.10]	0.14 [0.09; 0.26]	0.10 [0.03; 0.14]	0.19 [0.11; 0.31]
	p		**0.0179** *	0.7950***	0.0689** 0.7125****
CAR	Me [25; 75]	10.31·10^–6^ [10.31·10^–6^; 10.31·10^–6^]	11.55·10^–6^ [11.55·10^–6^; 11.55·10^–6^]	11.47·10^–6^ [11.47·10^–6^; 11.48·10^–6^]	12.73·10^–6^ [12.73·10^–6^; 12.73·10^–6^]
	p		**0.0001** *	**0.0001** ***	**0.0001** ** **0.0001** ****
dNLR	Me [25; 75]	0.30 [0.24; 0.33]	0.43 [0.40; 0.49]	0.31 [0.26; 0.41]	0.42 [0.36; 0.55]
	p		**0.0004** *	0.4697***	0.1057** 0.6646****
ALB/dNLR	Me [25; 75]	135.63 [112.91; 148.24]	86.93 [73.98; 104.77]	106.05 [84.85; 133.05]	78.51 [59.55; 94.69]
	p		**0.0003** *	0.1600***	0.0597** 0.4776****
NC/LC	Me [25; 75]	0.23 [0.20; 0.25]	0.30 [0.29; 0.33]	0.24 [0.21; 0.29]	0.29 [0.26; 0.35]
	p		**0.0004** *	0.5231***	0.1050** 0.6849****

N o t e: statistically significant differences between groups:

* control LR and experimental LR;

** control HR and experimental HR;

*** control LR and control HR;

**** experimental LR and experimental HR. LR — low-resistant; HR — high-resistant.

## Discussion

Synovial hypoxia, being a consistent RA sign, activates mechanisms that enable the body to adapt to an imbalance between tissue oxygen demand and oxygen supply. HIF-2α is a subunit of the hypoxia-inducible transcription complex HIF. It is expressed in fibroblast-like synoviocytes of RA patients and plays a fundamental role in disease pathogenesis. By regulating the inflammatory process, HIF-2α potentiates increased expression of the nuclear factor NF-κB receptor ligand, which controls the expression of genes involved in immune response regulation, apoptosis, and the cell cycle. It also induces several catabolic factors and activates the proliferative and osteoclastogenic potential of pannus cells [36].

In low-resistant rats, which have a genetically predetermined high level of HIF-1α produced in response to hypoxic environmental conditions, there is greater activation of the cellular immune system. This is mediated by HIF-1α-driven higher expression of NF-κB genes, leading to increased production of adhesion molecules and chemokines, as well as enhanced migration and reduced numbers of neutrophils undergoing apoptosis [37, 38]. Experimental evidence for the HIF involvement in RA pathogenesis was shown by Huh et al. [39] in a mouse model of arthritis: HIF-2α induction in chondrocytes contributed to cartilage erosion as it promoted chemokine production, which stimulated synovial fibroblast migration and invasion.

Our study provides histological confirmation of the additive and synergistic interaction between NF-κB and HIF, which resulted in a greater amplitude of inflammatory processes in LR animals compared to HR animals. Significant pathomorphological changes of the connective tissue were observed in 6 out of 12 LR individuals, while no such changes were observed in the HR group. Conversely, 7 out of 12 HR rats had only minor pathology, which was 2.3 times less frequent in the LR group (3 out of 12).

The total pathomorphological score for joint tissue changes was 1.5 times higher in LR rats than in HR rats (164 points/113 points, p≤0.05). General inflammation parameters, including joint cavity infiltrate, were 1.4 times higher (37 points/26 points, p≤0.01). A similar tendency was observed for following parameters: joint space narrowing (25 points/19 points), synovial hyperplasia (31 points/25 points), and pannus formation (36 points/26 points).

In RA, cartilage destruction is associated with increased expression of matrix metalloproteinases (MMPs), whose activity is regulated by endogenous tissue inhibitors (TIMPs). Hypoxia increases the expression of MMP-1 and MMP-3 in rheumatoid synovial fibroblasts, but decreases TIMP-1 expression at both protein and mRNA levels [40]. Researchers have demonstrated a significant role for HIF-1α in hypoxia-induced IL-8, MMP-1, and MMP-3 expression in fibroblast-like synoviocytes in RA [41]. Consequently, an increased MMP-1/MMP-3 to TIMP-1 ratio may lead to more aggressive joint destruction as tissue hypoxia intensifies during systemic inflammation in RA. Our findings indirectly support these data: histological examination of rat tarsal and metatarsophalangeal joints showed more severe cartilage destruction in low-resistant animals compared to high-resistant ones. The severity of cartilage degeneration was 1.6 times higher in LR rats than in HR rats (29 points/18 points, p≤0.01).

Poubelle et al. [42] found that neutrophils from synovial fluid expressed the receptor activator of nuclear factor-κB ligand, being an osteoclast differentiation factor initiating bone resorption. Therefore, greater inflammatory intensity involving neutrophils may underlie the increased osteolysis observed in LR animals. Our histological analysis confirmed this hypothesis: bone tissue changes were observed only in LR rats with RA (osteolysis — 6 points, p≤0.01).

Given the pathogenetic RA mechanisms, formulas for calculating inflammation biomarkers should account for the role of the cellular immune system. The MLR index represents the ratio of monocytes (the inflammatory component) to the indicators of the immune system’s regulatory mechanisms (T and B lymphocytes, and natural killers in total). The MLR index was less informative than the SIRI systemic inflammation index (which also includes the neutrophil count). We established approximate reference ranges for SIRI in healthy animals based on hypoxia resistance (0.07 to 0.10 units) and at day 35 of disease (LR: 0.14 units, p≤0.01; HR: 0.19 units, p>0.05) (see Table 3). These values correlated with histological disease severity in animals with high and low hypoxia resistance.

According to the literature data, MLR can effectively predict cardiovascular disease progression [33]. In our experiment, MLR did not differentiate between LR and HR animals in the control group (0.05 units), and although experimental group MLR values (LR/HR: 0.06 units/0.10 units) showed a trend, they did not reliably reflect the reference range of inflammation intensity or correspond to identified histologically graded pathomorphological features of RA.

In a study by Muravyov et al. [29], the calculated neutrophil-to-lymphocyte ratio (NLR) in RA patients correlated with DAS28, CRP, and ESR, unlike the platelet-to-lymphocyte ratio. In our study, the predictive value of NC/LC index was minimal, as it did not reflect disease dynamics in animals with different hypoxia resistance.

As the cellular immune system (neutrophils, lymphocytes) plays an important role in RA pathogenesis, we calculated the derived neutrophil-to-lymphocyte ratio — dNLR. dNLR has been reported to be a negative prognostic marker in various systemic diseases, including malignant tumors, multiple sclerosis, and sepsis [43, 44]. Independent research groups monitoring RA patients showed that dNLR levels were elevated compared to healthy controls and correlated with acute-phase reactants, DAS28, clinical signs, and ultrasound findings [28, 45–47]. In our experiment, dNLR did not significantly differ between healthy animals with different hypoxia tolerance (LR/HR: 0.30 units/0.31 units), but indicated inflammation in both experimental groups (LR/HR: 0.43 units/0.42 units). As the coefficient did not reflect the severity of pathomorphological changes in LR and HR animals, we incorporated an additional parameter essential in RA pathogenesis — serum albumin.

Albumin is involved in inflammation and acts as an immune system modulator. It influences antibody transport, immune complex formation, and overall immune response efficacy; it plays an important role in transporting hormones, metabolites, and drugs; it maintains normal oncotic pressure; and it contributes to antioxidant defense [48]. During RA exacerbation, albumin levels decrease due to its increased consumption at inflammation sites, while they tend to increase during remission. Albumin also regulates humoral immunity [49]. Under inflammatory conditions, oxidized albumin activates monocytes and neutrophils [50] and promotes p38 MAPK-mediated inhibition of pro-inflammatory cytokine production via TLR3 and TLR4 signaling pathways. It should be noted that TLR3 and TLR4 activate the transcription factor NF-κB, whose dysregulation potentiates inflammation, induces pro-inflammatory cytokines (type I interferons), initiates autoimmune processes, and contributes to cancer development [51–53]. Albumin also influences connective tissue histoarchitecture, with fibroblasts and extracellular matrix (composed of proteins such as collagen, albumin, globulins, and carbohydrates such as glycosaminoglycans) being key components. The extracellular matrix is formed through collagen and glycosaminoglycan production by fibroblasts, as well as plasma-derived components such as albumin and globulins [54]. Therefore, serum albumin levels are relevant for assessing pathomorphological changes in joint tissues in RA.

In our experiment, the ALB/dNLR index had reference ranges of 106.05 units (HR) to 135.63 units (LR). In the experimental groups, values were lower than in controls (LR/HR: 86.93 units/78.51 units, p≤0.01) and correlated with histological connective tissue pathology parameters. Thus, the integral inflammation index ALB/ dNLR may be recommended for RA prognosis. Notably, in a study by Ganeb et al. [49] monitoring RA patients during treatment, ALB/dNLR values were higher in patients than in healthy controls. This discrepancy likely reflects the high sensitivity of albumin concentration to drug therapy, as albumin actively participates in drug transport. The decreased index in our study aligns with both physiological and mathematical logic, as, according to the ALB/dNLR calculation formula, serum albumin concentration (which decreases during RA inflammation) is in the numerator, while the inflammatory dNLR index (which increases) is in the denominator.

CRP is one of standard laboratory diagnostic criteria for RA. As a key acute-phase mediator, CRP increases significantly (up to 1000-fold) at sites of infection, trauma, ischemia, and inflammation. It allows to monitor the disease activity over time. Persistently elevated CRP may indicate subclinical autoimmune diseases, intestinal and joint inflammation, or atherosclerosis [55]. Although not specific to RA, CRP is important for disease monitoring as it reflects the presence and severity of connective tissue inflammation. High CRP levels at treatment initiation and during treatment are considered poor prognostic indicators and vary with age and disease severity [56].

Both negative (albumin) and positive (CRP) acute-phase proteins are synthesized in the liver, where the majority of amino acids are used for positive acute-phase protein synthesis rather than albumin [57].

Given these considerations, the CRP-to-albumin ratio (CAR) may have greater prognostic value in RA. Our results support this hypothesis: CAR showed statistically significant reference ranges in both healthy animals (LR/HR: 10.31·10^–6^ units/11.47·10^–6^ units; p≤0.0001) and RA rats with genetically determined high and low hypoxia resistance (LR/HR: 11.55·10^–6^ units/12.73·10^–6^ units; p≤0.0001; r_xy_=0.57). The index also demonstrated statistically significant differences within each control–experimental group pair (p≤0.0001) and correlated inversely with histologically confirmed RA severity.

Thus, the results of this study showed that the severity of pathomorphological RA changes — specifically the severity of cartilage degeneration (LR/HR: 29 points/18 points, p≤0.01), general joint inflammation (LR/HR: 37 points/26 points, p≤0.01), and osteolysis (LR/HR: 6 points/0 points, p≤0.01) — was most effectively reflected by the following integral inflammation indices: CAR (LR/HR: 11.55·10^–6^ units/12.73·10^–6^ units; inverse correlation: r_xy_=0.57), ALB/dNLR (LR/HR: 86.93 units/78.51 units), and SIRI (LR/HR: 0.14 units/0.19 units).

## Conclusion

The assessment of the diagnostic potential of the new systemic inflammation biomarkers MLR, SIRI, CAR, dNLR, and ALB/dNLR showed that CAR was the most accurate for predicting the severity of RA manifestations, significantly reflecting the extent of pathomorphological joint changes in induced RA, while accounting for the organism's resistance to hypoxia. The integrated inflammation indices ALB/dNLR and SIRI may be recommended for individual monitoring of connective tissue pathology in RA. The prognostic value of MLR and NC/LC was low. The results of this study may be used to optimize the diagnosis, treatment, and prevention of RA, taking into account individual patient characteristics.
